# Antibiotic prophylaxis in ragged placental membranes: a prospective, multicentre, randomized trial

**DOI:** 10.1186/s12884-019-2373-9

**Published:** 2019-07-11

**Authors:** Hian Yan Voon, Jun Yan Pow, Lee Na Tan, Haris Njoo Suharjono, Wan Sim Teo

**Affiliations:** 1 Department of Obstetrics and Gynaecology, Sarawak General Hospital, Hospital Road, 93586 Kuching, Sarawak Malaysia; 2 Department of Obstetrics and Gynaecology, Sri Aman Hospital, Hospital Road, 95000 Sri Aman, Sarawak Malaysia; 3 Department of Obstetrics and Gynaecology, Bintulu Hospital, Nyabau Road, 97000 Bintulu, Sarawak Malaysia; 4 Department of Obstetrics and Gynaecology, Faculty of Medicine and Health Sciences, Universiti Malaysia Sarawak, 94300 Kota Samarahan, Sarawak Malaysia

**Keywords:** Antibiotic, Endometritis, Placental membranes, Prophylaxis, Ragged membranes

## Abstract

**Background:**

Ragged placental membranes is a distinct entity from retained placenta and not uncommonly reported in midwifery texts. Although the incidence of postpartum endometritis is merely 1–5% after vaginal births, it remains the most common source of puerperal sepsis, contributing up to 15% of maternal mortality in low income countries. Geographically-remote centres in Malaysia prophylactically administer antibiotics for women with ragged placental membranes after vaginal birth, extrapolating evidence from retained placenta. We sought to clarify the rationale in continuing such practices.

**Methods:**

This was an open-label, prospective, multicentre, randomized trial. Three hospitals where the current protocol was to administer prophylactic amoxycillin-clavulanic acid served as the sites of recruitment. Women who delivered vaginally beyond 24^+ 0^ weeks of gestation with ragged membranes were invited to participate in the trial and randomized into prophylaxis or expectant management with medical advice by blocks of 10, at a 1:1 ratio. A medication adherence diary was provided and patients followed up at 2 weeks and 6 weeks postpartum.

**Results:**

A total of 6569 women gave birth vaginally in three centres during the trial period, of which 10.9% had ragged membranes. The incidence of endometritis was not significantly raised in women with or without prophylaxis (0.90% vs 0.29%; *p* = 0.60). All cases of endometritis presented within the first 2 weeks and preventive use of antibiotics did not ameliorate the severity of endometritis since rates of ICU admission, surgical evacuation and transfusion were comparable.

**Conclusion:**

Preventive use of antibiotics after vaginal delivery in women with ragged placental membranes did not result in a reduction of endometritis. Educating women on the signs and symptoms of endometritis would suffice. Based on the reported incidence of ragged membranes, a change in practice would result in 1500 less prescriptions of antibiotics per annum in these three centres.

**Trial registration:**

NCT 03459599 (Retrospectively registered on 9 March 2018).

**Electronic supplementary material:**

The online version of this article (10.1186/s12884-019-2373-9) contains supplementary material, which is available to authorized users.

## Background

Puerperal sepsis is one of the leading direct causes of maternal mortality worldwide, as the process of childbirth inherently increases a woman’s exposure to infections arising from the genital tract. The 2014 MBRRACE-UK (Mothers and Babies: Reducing Risk through Audits and Confidential Enquiries across the UK) report highlighted an unenviable fact that 25% of women who died had succumbed to sepsis [1]. While postpartum haemorrhage, obstetric embolism and hypertensive disorders remain the principal causes, an unsettling trend is also observed locally, with maternal deaths from puerperal sepsis doubling from 1.6 to 3.1% in the latest Malaysian Confidential Enquiries into Maternal Deaths [2].

Although the incidence of postpartum endometritis is merely 1–5% after vaginal births, it remains the most common source of puerperal sepsis [3, 4]. It is estimated that postpartum endometritis contributes 15% of maternal mortality in low income countries. Long term complications from postpartum endometritis such as chronic pelvic inflammatory disease, ectopic pregnancy and subfertility not only lead to repeated health care visits but are substantial morbidities in survivors [5]. Among the reported risk factors for postpartum endometritis, prolonged rupture of membranes, use of internal fetal monitoring, operative vaginal delivery and caesarean section can be found. However, other than caesarean section, these risk factors did not appear to be significant predictors of subsequent infection [6].

Ragged placental membranes is a distinct condition from retained placenta or placental cotyledon and is often cited in midwifery texts. Its clinical significance lies in the potential for uterine subinvolution, haemorrhage and infection [7, 8]. The presence of ragged membranes is routinely documented in delivery notes and codable by the International Classification of Diseases, 10th Revision (ICD-10), [9]. The literature reflects a distinct paucity of data specifically relating to ragged or retained membranes, resulting in the authors extrapolating information from prophylactic antibiotic use in proxy conditions such as retained placenta, operative vaginal delivery and even routine postnatal prophylaxis as described below.

The World Health Organization (WHO) recommends the use of antibiotics prior to manual removal of placenta but acknowledges the lack of randomized controlled trials to support it, as evidence is largely extrapolated from caesarean sections [10, 11]. Indeed the lack of data is surprising, given that it is a common obstetric complication. Chongsomchai and colleagues have not been able to identify any studies in their systematic review, back in 2006 and as recent as 2014 [12]. A recent meta-analysis of retrospective cohort studies was performed in an attempt to clarify this, showing a non-significant reduction of endometritis with prophylaxis (OR 0.84), although the definition of endometritis was not explicit and the results did not achieve statistical significance [13].

Interestingly, the use of antibiotic prophylaxis for women who deliver vaginally has also been examined in women after instrumental delivery, routinely after any vaginal delivery and even antenatally in women with risk factors for sexually transmitted disease [14–16]. In all the scenarios above, reductions in the incidence of endometritis were found. A Cochrane review identified one trial involving 393 women who required operative vaginal delivery, in which 7 developed endometritis without antibiotic prophylaxis while none had such complications in the treatment group. The trial was judged to be of low risk for bias but in the absence of additional evidence, the reviewers abstained from recommending prophylaxis [14].

A large French randomized trial recruited low risk women after vaginal delivery and administered a single dose of broad spectrum antibiotics before discharge. The authors found a four-fold reduction in endometritis with prophylaxis and were still able to demonstrate a 70% cost saving in such women, compared to subsequent treatment. The role of the pharmaceutical company was unclear and there was no disclosure by the authors [15].

Therefore, administering antibiotics to women adjudged to be at risk of endometritis, as evidenced by the presence of ragged membranes invites controversy. The hospitals invited to participate in this trial currently implement the protocol of giving amoxycillin-clavulanic acid as prophylaxis if ragged or retained membrane is present after vaginal birth. These hospitals serve a large patient catchment area in the Malaysian state of Sarawak and face considerably more logistical challenges than many hospitals in Peninsular Malaysia. While some of the hospitals serve as referral centres and are located in larger towns, the patients eventually return to more geographically-remote areas postnatally. This remains the principal reason behind the protocol of providing prophylaxis for many years, despite the lack of concrete evidence. An unpublished survey by the lead author reveals that similar practice does not apply in many hospitals in Peninsular Malaysia.

However, with significant improvements in rural health care facilities and telecommunications connectivity over the past decade, we wish to re-examine the role of continuation of such a protocol. Furthermore, finite financial resources and patient concerns about medicalization of obstetric care provides an additional impetus for this paper. Therefore the risk-benefit ratio of undertaking this trial is favourable.

## Methods

This was an open-label, prospective, multicentre, randomized trial. Three hospitals in Sarawak where the current local protocol was to administer prophylactic amoxycillin-clavulanic acid served as the sites of recruitment. Eligible women were randomized to receive either prophylaxis, as part of the existing protocol (control) or no prophylaxis (intervention). Both groups of women were given medical advice regarding symptoms of endometritis as detailed below. No modifications to the dose, timing or mode of administration of the intended medication were made to the prophylaxis group.

**Fig. 1:**
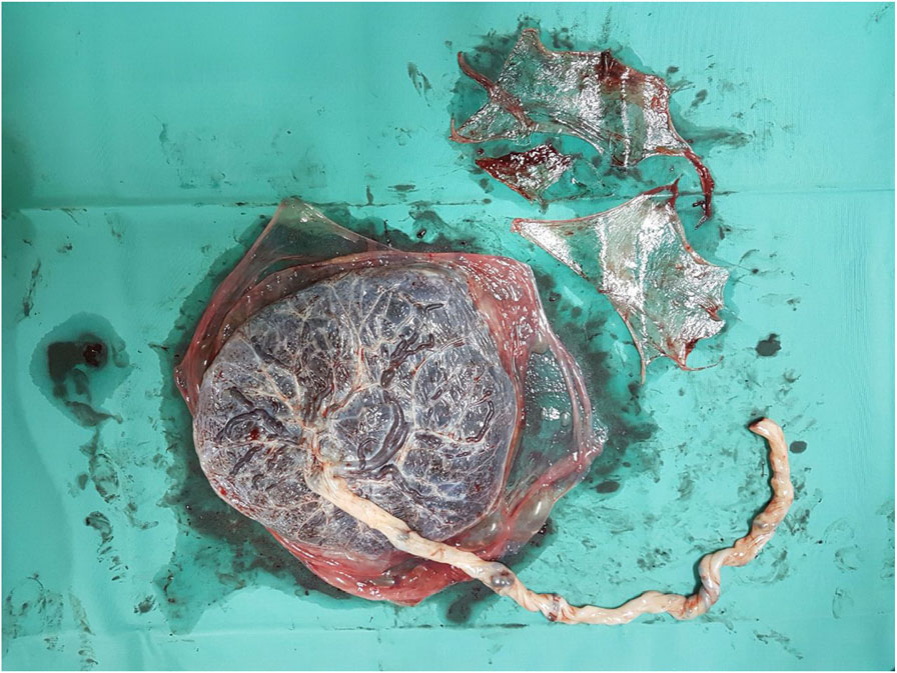
Examination of the placental surface of placenta. Ragged membranes were evacuated separately and shown on the top right corner

**Fig. 2:**
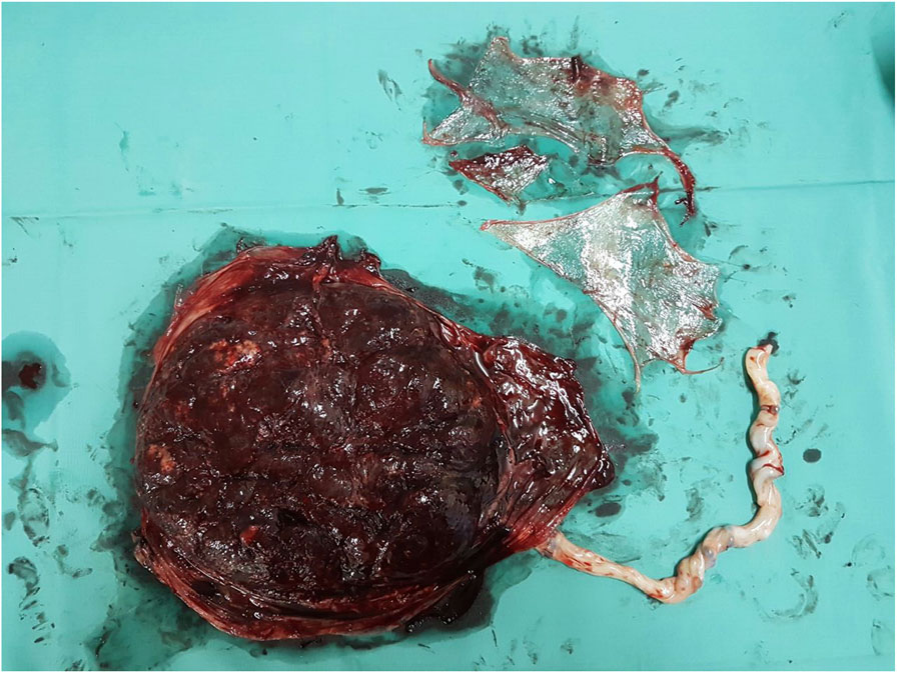
Examination of the maternal surface of placenta. Ragged membranes were evacuated separately and shown on the top right corner

Women who delivered vaginally beyond 24^+ 0^ weeks of gestation and were found to have ragged placental membranes were invited to participate in the trial. This was defined as placental membranes perceived to be incompletely removed by the accoucher during the third stage of labour, as opposed to delivery of membranes en bloc with the main placental mass. The appearance may involve irregular, scalloping edges of the membranes when inspected and is typically but not invariably, associated with pieces of membrane requiring evacuation from the cervical os (Figs. 1 and 2). When the completeness of placental membranes was in doubt, a second, more experienced accoucher would be consulted.

In all women where ragged placental membranes were diagnosed, immediate bedside digital evacuation from the dilated cervical os was attempted. Regardless of the amount of membranes removed, the diagnosis holds. However, if suspicion arose that a whole layer of chorionic or amniotic membrane was retained, formal assessment by the labour ward registrar was triggered.

Exclusion criteria were as follows:

- Fever, within 5 days preceding delivery (axillary temperature > 37.5 °C on 2 or more occasions at least 1 h apart or temperature > 38 °C on one occasion). This also includes intrapartum fever
- Required oral or intravenous antibiotics for any other obstetric-related (ex. third or fourth degree tears, preterm prelabour rupture of membranes) or non- obstetric related (ex. pneumonia, acute pyelonephritis) reasons
- Retained placenta
- Prolonged rupture of membrane (> 18 h)
- Retroviral disease, on long term oral or parenteral steroid or receiving other forms of immunosuppressants, including chemotherapy within the last one year
- Vaginal delivery for an intrauterine death
- Penicillin allergy

Recruitment took place in the labour suite of the respective hospitals, approximately an hour after delivery and perineal repair. The mothers were routinely counseled regarding red-flag symptoms suggestive of endometritis such as lower abdominal pain, increasing lochia loss and fever, as was the current practice. A patient information leaflet in either English or Malay was provided before they were transferred out of the labour suite. A second point of contact was made by investigators within 6–8 h to confirm their interest in participation. Written, informed consent were taken from women who agreed to participate before randomly assigning them on a ratio of 1:1 (prophylaxis arm or no prophylaxis arm) by block randomization (block size of 10), stratified by site of recruitment, using a web-based randomization program.

Women allocated to the prophylaxis arm received a week’s course of amoxycillin-clavulanic acid tablets 625 mg three times a day, with the first dose administered within 6–8 h after delivery. A patient diary was provided to enhance their adherence to medication. This is an open label trial and did not involve the use of placebo. The author performing the analysis (VHY) was not involved in recruitment and remained blinded to the assigned arms. Women who declined participation would be excluded from the trial and given prophylaxis as per local protocol and advised on the red-flag symptoms of endometritis.

Upon discharge, follow-up telephone calls were conducted by the authors at 2 weeks and 6 weeks post-delivery. Medication adherence, discontinuation and side effects would be clarified. Symptoms of endometritis were enquired and if present, women were seen within 24 h in the Obstetric Daycare Unit of the respective hospitals. The follow-up telephone call would also allow the detection of any admission for endometritis in these women, should they be admitted to hospitals other than the ones involved in recruitment.

Postpartum endometritis was defined as any intrauterine infection occurring after the third stage of labour until 6 weeks post delivery. At least 2 of the following symptoms or signs would have to be present at the time of diagnosis [5, 17]:

- Fever (axillary temperature > 37.5 °C on 2 or more occasions at least 1 h apart or temperature > 38 °C on one occasion), occurring in the absence of apparent source of infection or alternative foci of infection.
- Increasing lochia loss or offensive lochia.
- Lower abdominal pain or suprapubic tenderness on palpation.

Alternatively, in the event that only one of the symptoms above was present, the diagnosis of endometritis could also be established by:

i. Positive genital swab culture in the presence of (a).
ii. Elevated total white cell count > 11.0 × 10^9^ cells/L in the presence of (b) or (c).

Women who developed endometritis were treated as per the local hospital’s protocol, at the discretion of the attending healthcare provider. This included outpatient treatment, admission for intravenous antibiotics or surgical evacuation. No additional serum monitoring for transaminitis was warranted for amoxycillin-clavulanic acid as this risk is low and there are no recommendations from the manufacturer. There were no changes to the methods after trial commencement. All information collected were transcribed to a pre-piloted proforma by the respective site coordinators. A single hard copy was kept in a designated locked compartment within the recruitment site, accessible only to the site coordinator and compiled into an electronic format at the end of the period of follow up. The electronic copy was devoid of patient identifiers. Once the information in the electronic copy was deemed to be complete and cross checked by coordinators from another site, the hard copy was destroyed as agreed upon during the ethical approval process.

We designed this trial to establish a 5-fold reduction in the incidence of endometritis from 5% in the interventional group (no prophylaxis) to 1% in the control group (prophylaxis continued as per protocol). The baseline rate of endometritis and reduction of the event with prophylaxis were estimated from existing studies [4, 15, 18]. The power of the trial (1-beta) was set at 0.85 with a significance level (alpha) of 0.05. The sample size was calculated using PS software version 3.0.12 [19].

Three hundred twenty-five women on each arm would be required to reject the null hypothesis that antibiotic prophylaxis resulted in a five-fold reduction of postpartum endometritis after vaginal delivery in women who had ragged placental membranes.

Analysis was performed using SPSS 21.0™ (IBM). Descriptive analysis would be performed on patient demographics. Incidences of the primary outcomes (postpartum endometritis), secondary outcomes (ICU admission, surgical evacuation of products of conception and blood transfusion) were calculated. Categorical data were analysed using Chi square test and continuous data with independent T-test. Continuous variables which were not normally distributed, such as the duration of second stage were analysed using Mann-U Whitney test. Analysis was by intention-to-treat.

Approval from the Medical Research and Ethics Committee (MREC), Ministry of Health Malaysia was obtained prior to embarking on the trial (NMRR ID 16–1016-31,034). The trial was retrospectively registered with ClinicalTrials.gov (NCT 03459599).>

## Results

**Fig. 3:**
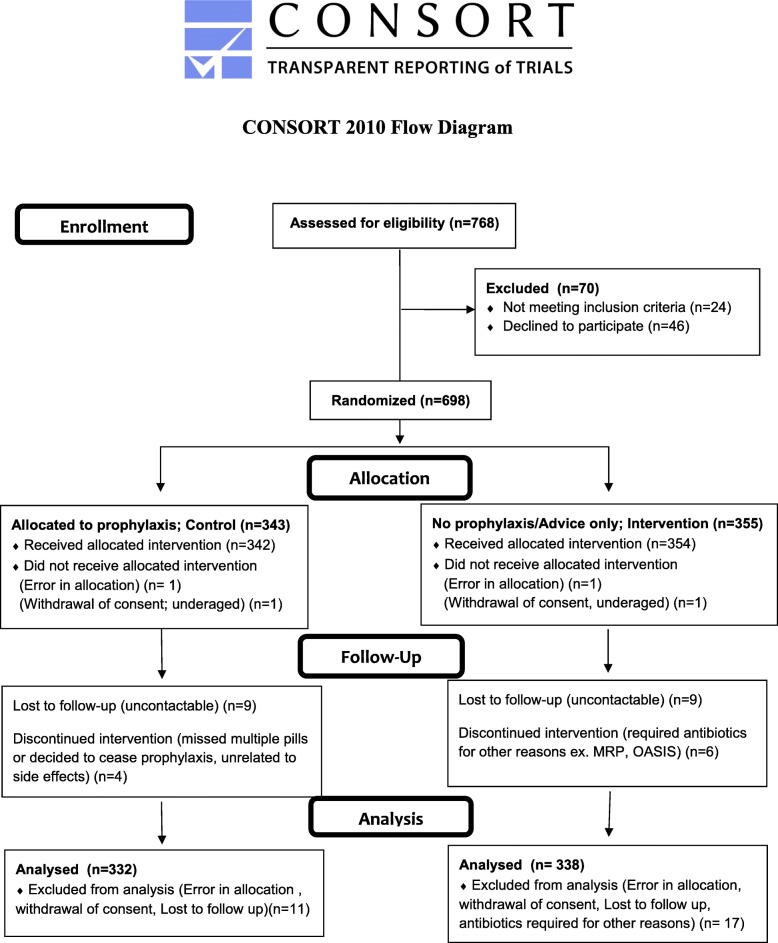
Enrollment, randomization and follow up

A total of 6569 women delivered vaginally across three centres during the trial period, from March to September 2017, of which 10.9% had ragged membranes. Recruitment was stopped when the required sample size was achieved. Figure 3 shows the CONSORT flow diagram from recruitment till analysis. A total of 670 patients were available for analysis, whereby 332 received prophylaxis and 338 did not. 11 women were excluded in the prophylaxis arm due to error in allocation, withdrawal of consent or loss of follow up. 17 women from the no prophylaxis arm were excluded for similar reasons but also included 6 women who required antibiotics due to other medical or obstetric indications. Sarawak General Hospital, Bintulu Hospital and Sri Aman Hospital contributed 577 (86.1%), 61 (9.1%) and 32 (4.8%) patients respectively, proportional to the delivery delivery rates of each centre.

There were no differences between both groups in terms of age, parity, body mass index at booking, history of smoking, sexually-transmitted disease or previous preterm labour prior to 34 weeks. Mean gestational age at delivery, instrumental delivery, median second stage duration and the number of vaginal examinations were comparable (Table 1). Five women required internal fetal scalp electrode, all of whom were from the group that did not receive prophylaxis, while there were none in the prophylactic group.

**Table 1:** Baseline and intrapartum characteristics of trial participants

Characteristics	Prophylaxis *n* = 332	No prophylaxis *n* = 338	*P*-value
Baseline			
Age (years)	28.04 (5.67)	28.00 (6.02)	0.94
Parity (number)	2.42 (1.30)	2.40 (1.48)	0.83
BMI (kg/m^2^)	25.15 (5.22)	24.51 (5.18)	0.11
Smoking	7 (2.1%)	4 (1.2%)	0.38
Previous preterm < 34 weeks	10 (3.0%)	8 (2.4%)	0.60
Sexually transmitted disease	0 (0.0%)	0 (0.0%)	–
Intrapartum			
Gestation at delivery (weeks)	38.63 (1.68)	38.61 (1.67)	0.84
Internal fetal monitoring	0 (0.0%)	5 (1.48%)	0.06**
Instrumental delivery	16 (4.80%)	19 (5.20%)	0.64
Second stage duration (min)*	7	8	0.07
Number of vaginal examinations	4 (2)	4 (2)	0.34

Values represent mean (standard deviation) or as number (percentages)

*Represent median value

**Fisher exact test used

The incidence of endometritis was not significantly raised in women with or without prophylaxis (0.90% vs 0.29%, *p* = 0.60). All women diagnosed with endometritis based on the predefined criteria, presented within the first two weeks after delivery. Women in the prophylactic group presented between days 8–13 while the sole patient without prophylaxis presented much earlier, on day 2.

Based on the diary kept by women who received prophylaxis, two of them were compliant while another missed six doses of medication. The latter felt that prophylaxis was unnecessary since she had been feeling well several days post delivery. No serious adverse effects were reported by patients receiving prophylaxis.

Intensive care unit admission, the need for blood transfusion and surgical evacuation were similarly low in both groups (Table 2). The average length of stay was three days.

**Table 2:** Outcomes of patients with ragged membranes

Complication	Prophylaxis *n* = 332	No prophylaxis *n* = 338	*P*-value
Endometritis	3 (0.90)	1 (0.29)	0.60
ICU admission	0	0	
Blood transfusion	1 (0.30)	1 (0.29)	0.32
Surgical evacuation	1 (0.30)	0	0.50

Values represent number (percentages); *ICU* Intensive care unit

## Discussion

This trial was conducted as part of the state’s on-going effort in keeping abreast with contemporaneous, evidence-based medicine, taking into account the improved accessibility of healthcare services. Such measures included elimination of unnecessary practices, videlicet antibiotic prophylaxis in ragged placental membranes.

The incidence of endometritis in our trial was similarly low regardless whether prophylaxis was given or not and the presentation of such cases was usually within the first fortnight post delivery. Although women who received prophylaxis appeared to present later, we were unable to conclude whether prophylaxis did in fact delay the presentation of endometritis due to the small number of women developing complications. The severity of endometritis did not appear to be ameliorated by the use of prophylaxis as there were no statistically significant differences in ICU admission, blood transfusion or need for surgical evacuation.

The findings in this trial was in contrast to the potential reduction in endometritis in other scenarios post-vaginal delivery, such as retained placenta and instrumental delivery described earlier [13, 14]. Interestingly, the role of *antenatal* antibiotic prophylaxis to prevent postpartum endometritis has also been studied in several randomized controlled trials. In fact, a systematic review and meta-analysis showed a 50% reduction in endometritis with prophylaxis, given between 26 and 32 weeks, although two thirds of the population included were deemed to be at high risk for sexually transmitted diseases [16]. However, it must be emphasized that such interventions were performed before the publication of the Oracle Children Study II in 2008. This landmark trial assessed children at the age of seven, showing an increased risk of cerebral palsy when their mothers were prescribed antibiotics for preterm labour and intact membranes [20]. Suffice to say, the attitude of obstetricians towards prescription of antibiotics antenatally has shifted since its publication. Furthermore, the incidence of sexually transmitted disease based on self-reported history or a positive serological or genital swab was low in our cohort.

One contentious aspect of this trial was our consensus to use the term “prophylaxis” rather than “treatment”, when in fact a full course of antibiotics was prescribed. The authors felt that treatment should be reserved for a recognized pathology rather than conditions of equipoise such as this. The reviewers aptly suggested we consider the term “preventive” use of antibiotics.

With approximately 13,500 vaginal deliveries per annum across these centres, it is estimated that a change in practice would see 1500 less women requiring superfluous treatment. This supports antimicrobial stewardship initiatives in preventing the emergence of antibiotic-resistant infections. Another area of concern with a more liberal policy on antibiotics is the alteration of neonatal gut microbiome with peripartum administration of antibiotics in breastfeeding women [21].

The strength of this trial included the prospective randomized design and a system for recall which allowed patients to be traced even if they were admitted to another institution during the trial period for endometritis. We were also able to monitor the compliance to medication in the prophylaxis arm. On the other hand, the incidence of endometritis was much lower than previously estimated. It was unlikely that the 18 patients lost to follow up, equally distributed between both arms, would contribute to the overall incidence of endometritis. Consequently, a much larger sample size would be required for statistical power. However, the authors felt that there was already sufficient evidence for cessation of such prophylactic practices locally and prolonging the trial would not be the best use of existing resources. From a broader perspective, a low complication rate in women with ragged membranes overall, calls into question whether this finding should perturb the clinician.

The potential risk of bias due to a lack of placebo would have minimal impact on the primary outcome, since endometritis was defined by objective criteria such as the presence of fever, increased vaginal losses, leukocytosis and a positive genital culture.

## Conclusion

Preventive use of antibiotics after vaginal delivery in women with ragged placental membranes did not result in a reduction of endometritis, ICU admission, transfusion or surgical evacuation. Advising women on the signs and symptoms of endometritis would suffice. In addition, the unnecessary use of antibiotics propagates medicalization of pregnancy, potentially alters fetal gut microbiome and should therefore be considered harmful.

## Additional file

Additional file 1: Contains tabulated anonymized raw patient data in XLSX Worksheet format. (XLSX 64 kb)

## Data Availability

The dataset supporting the conclusions of this article is included within the article and as additional files (Additional file 1).

## Abbreviations

ICU: Intensive Care Unit
kg: kilograms
m^2^: square metres
min:minute, NMRR: National Medical Research Register
WHO: World Health Organization
